# Functional Network Organization Underlying Individual Differences in False Feedback Susceptibility: A Graph Theoretical Analysis

**DOI:** 10.1002/hbm.70640

**Published:** 2026-10-30

**Authors:** J. Patchitt, T. Karapanagiotidis, S. N. Garfinkel, M. Miller, M. Tsakiris, A. Clark, H. D. Critchley

**Affiliations:** ^1^ Sussex Centre for Consciousness Science, University of Sussex Brighton UK; ^2^ Department of Clinical Neuroscience Brighton and Sussex Medical School, University of Sussex Brighton UK; ^3^ School of Psychology, University of Sussex Brighton UK; ^4^ Institute of Cognitive Neuroscience, University College London London UK; ^5^ Monash Centre for Consciousness and Contemplative Studies, Monash University Melbourne Victoria Australia; ^6^ Department of Psychology Royal Holloway, University of London Egham UK; ^7^ School of Media, Arts and Humanities, University of Sussex Brighton UK; ^8^ School of Engineering and Informatics, University of Sussex Brighton UK

**Keywords:** emotion recognition, false physiological feedback, graph theory, interoception, perception, predictive coding, resting state

## Abstract

Individual differences in susceptibility to somatosensory false physiological feedback reflect variations in interoceptive processing, yet the underlying neural network mechanisms remain unclear. We analyzed resting‐state functional connectivity using graph theoretical approaches in 41 healthy adults who completed a somatosensory false feedback paradigm. Nine regions of interest were defined from previous activation analyses, and network topology metrics including local efficiency, clustering coefficient, centrality measures, and path length were calculated across multiple thresholds. Three distinct patterns of network organization predicted false feedback susceptibility. Individuals more susceptible to false feedback showed: (1) increased posterior insula eccentricity, indicating network peripheralization of this key interoceptive region; (2) consistent orbitofrontal cortex segregation across multiple metrics including increased path length and decreased centrality measures; and (3) cerebellar network reconfiguration with reduced local efficiency but increased betweenness centrality. Somatosensory false feedback susceptibility reflects specific alterations in functional network topology, particularly reduced integration of interoceptive regions (posterior insula) and emotional appraisal systems (orbitofrontal cortex). These findings support a network‐based model where interoceptive processing depends on efficient integration between visceral sensation, emotional evaluation, and cognitive control systems. Understanding these network properties may inform interventions targeting interoceptive awareness and emotional regulation.

## Graphical abstract

Individual differences in susceptibility to somatosensory false feedback (sFFB) predict altered network topography between regions previously implicated in sFFB. This work supports an interoceptive integration model of sFFB and highlights the importance of network topology in determining how individuals weigh and integrate internal and external physiological information during emotional processing.

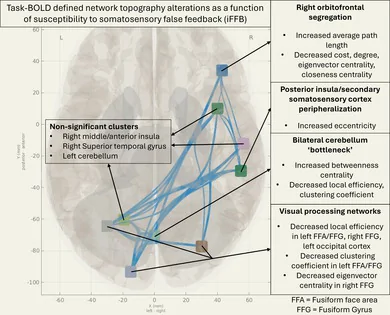

## 1 Introduction

Interoception is the sense of the internal state of the body and encompasses the body‐to‐brain signaling and central representation of physiological states. It provides crucial information for reactive homeostatic reflexes and predictive allostatic responses that enable survival through adaptive visceromotor, neuroendocrine and behavioral change. Interoceptive sensations underpin motivational and affective feelings to allow individuals to recognize and interpret their own bodily states, regulate emotions and select strategic behavior (Critchley et al. 2004). Internal bodily sensations range from ‘organ‐specific’ visceral sensations, for example, pounding heartbeats and churning stomach, system‐selective sensations such as breathlessness or temperature, on to more integrated experiences of physiological arousal, for example, fatigue, hunger and thirst (Quadt et al. 2018). Critically, both pre‐attentive and conscious representations of interoceptive signals influence other psychological processes, guiding the perception of external (exteroceptive) stimuli, shaping action tendencies, and gating encoding into memory (Critchley and Patchitt 2024).

Interoception is commonly conceptualized within a hierarchical predictive‐processing framework, wherein ascending interoceptive signals are ‘judged’ against descending predictions about bodily states (Allen et al. 2022; Owens et al. 2018). At higher levels, these predictions are integrated to model the intended/optimal state of the individual and are expressed though efferent autonomic drive and behavioral (motor) actions. Understanding the brain‐wise network properties underlying interoceptive control within this predictive framework is essential for elucidating how physiological signals are fundamentally integrated with emotional and cognitive processing (Kleckner et al. 2017; Smith et al. 2017).

False physiological feedback (FFB) paradigms offer a unique window into these processes by experimentally manipulating higher‐order perceptual appraisal of physiological states. Initially developed by Valins (1966) using auditory feedback, these paradigms have evolved to incorporate tactile modalities that may engage interoceptive networks more directly. Recent work using somatosensory FFB (sFFB) demonstrated bi‐directional effects on emotional judgments (Patchitt et al. 2025) that contrast with the unidirectional effects typically observed with auditory FFB (Valins 1966; Gray et al. 2007). Neuroimaging evidence of somatosensory FFB shows the engagement of cortical regions supporting interoceptive and cross‐modal representations of bodily state with regions implicated in social/affective judgments: The processing and perceptual influence of false feedback is supported across right posterior, middle, and anterior insula, right orbitofrontal cortex (rOFC), supramarginal gyrus (SMG), bilateral fusiform and right superior temporal gyri (Patchitt et al. 2025), indicating a potential divergence from cognitive appraisal of an external auditory signal to interoceptive integration of a false quasi‐interoceptive/somatosensory signal.

Resting‐state network properties have been previously associated with distinct dimensions of interoception. For instance, across individuals, functional connectivity between the right inferior frontal operculum (rIFO), middle temporal gyrus, and bilateral SMG positively correlates with self‐rated mind–body integration as measured by the Multidimensional Assessment of Interoceptive Awareness (MAIA) Scale (Stoica and Depue 2020). The functional organization within insular cortex changes topographically when attention is switched between interoceptive, exteroceptive, and emotional information (Simmons et al. 2013) and the profile of posterior insula functional connectivity during interoceptive (vs. exteroceptive) attention distinguishes individuals judged to be interoceptively ‘good’ and ‘bad’ from objective performance accuracy on a heartbeat detection task (Kuehn et al. 2016). Other studies similarly describe resting state network connectivity differences between people classified heuristically according to ‘conscious interoception accuracy’ from performance accuracy on adaptations of the heartbeat counting task (Schandry 1981; Chong et al. 2017). Limitations (e.g., unsurprising ‘top‐down’ cognitive biases) have been acknowledged for three decades (e.g., Ring and Brener 1996; Zamariola et al. 2018) yet the pragmatic use of this task continues to excite debate about definition and interpretation and attests to the desire to develop objective measures of how internal sensations are perceived (Murphy et al. 2019; Corneille et al. 2020; Desmedt et al. 2023). Nevertheless, there is growing neuroimaging evidence relating specific functional network properties to individual differences in how interoceptive information is represented for conscious access (Medford and Critchley 2010).

Consistent with the primacy of internal physiological regulation to adaptive behavior and ultimately survival, the engagement and interaction of viscerosensory insular cortex and visceromotor anterior cingulate cortex is critical for the predictive control of interoceptive state (Critchley 2005; Craig 2009; Seeley et al. 2007). By extension, the functional interconnectivity of these regions is central the concept of the ‘salience network’ as a wider distributed functional substrate for detecting behaviorally salient stimuli and coordinating resources in response to physiologically relevant information (Menon and Uddin 2010). Beyond insular and cingulate regions, the right orbitofrontal cortex (rOFC) plays an important role in integrating visceral signals with emotional evaluation and decision‐making processes (Rolls 2019), while the cerebellum contributes to interoceptive processing through its capacity to compare predicted sensory outcomes with actual feedback (Ito 2008; Adamaszek et al. 2017). Recent evidence has highlighted the cerebellum's participation in salience network functioning, with distinct cerebellar subnetworks showing functional connectivity to key interoceptive regions (Habas et al. 2009; Buckner et al. 2011). The cerebellum's role extends beyond motor coordination to include the integration of cognitive, affective, and physiological information, suggesting its involvement in detecting mismatches between expected and actual bodily states—a function particularly relevant in false feedback paradigms.

Network organization within salience‐related regions unfolds dynamically during periods of anxious anticipation, suggesting that interoceptive network properties are not static but can change with physiological and emotional states (McMenamin et al. 2014). Performance accuracy on heartbeat detection tasks is related to the strength of functional connectivity between the right posterior insula (rPI) and related salience network regions (Chong et al. 2017) and in older adults, between the left rostral prefrontal cortex, right insula, and rOFC (Ueno et al. 2020). Mapping such interoceptive network dynamics has important clinical implications for understanding the impact of neurodegeneration and stroke on emotion, behavior, and function (García‐Cordero et al. 2016).

The focus on resting‐state functional connectivity is predicated on substantial evidence that intrinsic network architecture reflects stable organizational principles that constrain and predict task‐evoked brain responses (Smith et al. 2009; Cole et al. 2014). Resting‐state networks closely correspond to task‐activation networks across multiple cognitive domains, suggesting that the same functional architectures observed during rest are recapitulated and modulated during active task performance (Smith et al. 2009; Tavor et al. 2016). From a predictive processing perspective, resting‐state connectivity patterns can be conceptualized as reflecting baseline priors—the brain's intrinsic functional organization that shapes how ascending interoceptive and exteroceptive information is weighted and integrated during active processing (Sadaghiani et al. 2009; Raichle 2015). Individual differences in these baseline network configurations may therefore determine the relative influence of internal versus external sources of physiological information when individuals are presented with conflicting signals, as occurs in false feedback paradigms. Examining resting‐state topology thus provides insight into the stable network properties that predispose individuals to differential susceptibility to false feedback, independent of state‐dependent fluctuations during the task itself (Finn et al. 2015; Gratton et al. 2018).

Graph theoretical approaches to analyzing resting‐state functional connectivity provide powerful tools for understanding the network organization underlying distributed processing across the brain (Bullmore and Sporns 2009; Rubinov and Sporns 2010). These methods characterize complex brain networks in terms of their structural and functional properties, including local efficiency, clustering, centrality, path length, and eccentricity (Fornito et al. 2016; Farahani et al. 2019). Each metric offers distinct insights: local efficiency reflects the potential for information transfer within subnetworks (Latora and Marchiori 2001); clustering coefficient quantifies local integration (Watts and Strogatz 1998); centrality can identify hub regions critical for information flow (van den Heuvel and Sporns 2013); and path length and eccentricity assess network integration and segregation (Achard and Bullmore 2007). In the context of interoception, these graph theoretical metrics can reveal how the integration and segregation of brain networks influence an individual's processing of physiological signals (Bassett and Sporns 2017; Betzel and Bassett 2017).

This investigation utilized graph theoretical approaches to analyze resting‐state functional connectivity in relation to individual susceptibility to somatosensory false feedback (iFFB) in a sample previously investigated in Patchitt et al. (2025). We hypothesized that distinct patterns of network organization, particularly in interoceptive regions such as the posterior insula, anterior insula, orbitofrontal cortex, cerebellum, and supramarginal gyrus, would predict susceptibility to false feedback effects. Specifically, we predicted that alterations in network efficiency, integration, and centrality in these regions would be associated with increased sensitivity to false physiological feedback.

## 2 Methodology

### 2.1 Participants

We recruited 41 non‐clinical adult volunteers between 18 and 65 years old, self‐declared free from illness, disability, and psychoactive medication with normal or corrected‐to‐normal vision (i.e., glasses, contact lenses, laser eye surgery). No excessive consumption of alcohol (< 10 glasses per week), caffeine (equivalent of 2 cups of regular coffee per day) or any illegal drugs 2 months prior to the study. Additional criteria for the fMRI session included no irreversibly attached metal in the body, claustrophobia, dermal plates, or contraceptive coils. Ethical approval was granted by Brighton and Sussex Medical School, Research, Governance and Ethics Committee: BSMS9NYX/1.

### 2.2 Procedure

Eligible participants visited the Clinical Imaging Sciences Centre at the University of Sussex, provided written consent and were then given the State Trait Anxiety questionnaire, Multidimensional Assessment of Interoceptive Awareness questionnaire, and Becks Depression Inventory. A 3‐lead ECG was then fitted to the participants' chest in an L shape around the left breast, alongside a pulse oximeter on the left hand and a false feedback device on the right. The response button box was also placed in the right hand of the participant. First, 12 min of resting state images were acquired, followed by 1 min of spin echo field maps for susceptibility distortion correction. After which a 23‐min fMRI BOLD task was ran.

During fMRI BOLD acquisition, participants performed a simple emotional intensity rating task in which 80 faces were shown and repeated for all four conditions.

Conditions included false physiological feedback at (1) higher than participant's veridical HR (Higher FFB), (2) lower than participant's veridical HR (Lower FFB), (3) at participant's veridical HR, (4) no physiological feedback at all. These four conditions were randomly cycled throughout the experiment 16 times each, changing every 20 s for a total of 64 blocks. This gave participants 5 intensity rating trials per condition, resulting in 320 subjective intensity ratings. For more information on this task and the stimuli used see Patchitt et al. (2025). The experimental paradigm is illustrated in Figure 1 (reproduced from Patchitt et al. 2025).

**FIGURE 1:**
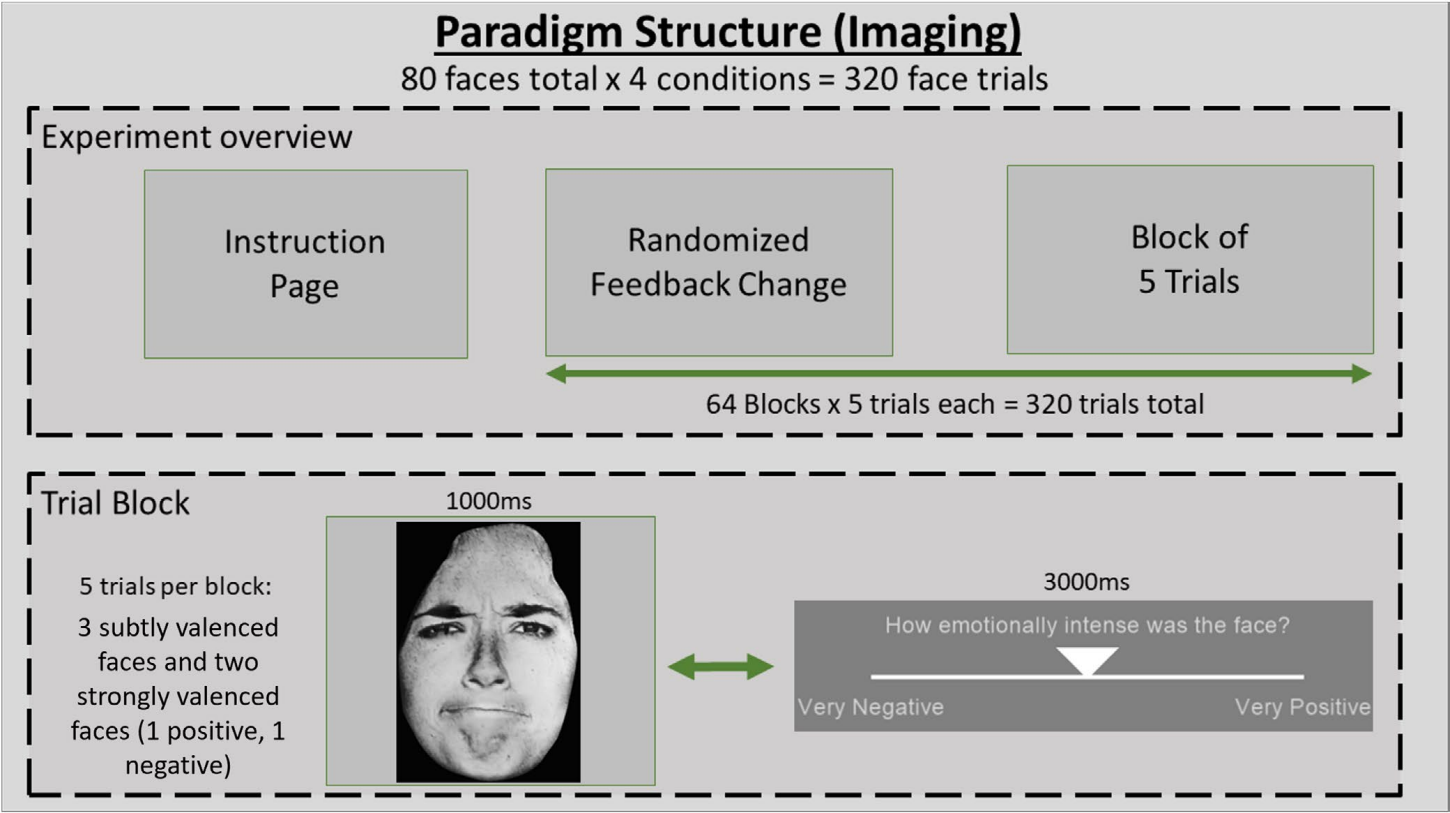
Visualization of fMRI BOLD task from Patchitt et al. (2025). Face stimuli (1000 ms) and rating screen (3000 ms) alternate between each other with Feedback condition changing every 5 iterations for a total of 64 blocks. Each block was pseudorandomized to ensure subjects were exposed to 3 expressions very close to neutral but slightly positively/negatively valenced (labeled neutral) and 2 strongly valenced expressions (labeled positive/negative).

After the fMRI BOLD sequence, a 7‐min NODDI diffusion‐weighted imaging sequence set was acquired that is analyzed elsewhere, followed by T1w and T2w structural image sequences. Once participants were out of the scanner, they were debriefed and received payment for participation.

### 2.3 Individual FFB Sensitivity Calculation

To quantify individual sensitivity to false cardiac feedback during negative emotion trials, we employed a trial‐by‐trial slope analysis. Data were first filtered to include only trials presenting negative emotional stimuli. This was noted by Patchitt et al. (2025) as being the emotional condition in which sFFB had the most notable effect. For each participant and feedback condition, linear regression models were fitted with trial number as the predictor and behavioral response as the outcome variable. The regression slope coefficient for each participant‐condition combination was extracted as an index of the response slope across trials within blocks.

Raw slope values were standardized across participants using *z*‐score transformation to account for individual differences in baseline response patterns. To isolate condition‐specific effects of false feedback, we computed a proportional mismatch index for each participant:

$$\begin{array}{c} \text{Mismatch}=\left(\text{Slope}\_\text{Higher}\_\text{sFFB}-\text{Slope}\_\text{Lower}\_\text{sFFB}\right)\\ \;/\left(\left(\text{Slope}\_\text{Higher}\_\text{sFFB}+\text{Slope}\_\text{Lower}\_\text{sFFB}\right)/2\right) \end{array}$$

This metric quantifies the relative difference in exposure effects between feedback conditions while normalizing for each participant's overall exposure effect magnitude.

Finally, mismatch scores were mean centered across the sample by subtracting the group mean, yielding a distribution centered at zero. This mean‐centered mismatch index served as our participant‐level measure of differential sensitivity to false cardiac feedback during negative emotion processing, suitable for subsequent correlation analyses with neuroimaging metrics.

All behavioral analyses were performed in R (version 4.3.2) using the tidyverse, dplyr, and broom packages.

### 2.4 fMRI Data Acquisition and Preprocessing

For fMRI and resting state scanning parameters see Tables 1 and 2 respectively.

#### 2.4.1 fMRI BOLD Scanning Parameters

**TABLE 1:** Scanning parameters for the fMRI BOLD sequence used in Patchitt et al. (2025).

Manufacturer	Siemens
Field strength	3T
Model name	Prisma
No. of sessions	1
Volumes acquired	899
Pulse sequence type	Gradient echo, EPI
Field of view	208 × 208 mm
Matrix size	104 × 104 × 72
Slice thickness	2 mm
No. of slices	72
Acquisition orientation	Axial
Whole brain	Yes
Slice acquisition order	Interleaved
TE	28 ms
TR	1520 ms
Multi‐band factor	3
Flip angle	75°

#### 2.4.2 Resting State fMRI Scanning Parameters

**TABLE 2:** Scanning parameters for the fMRI resting state sequence.

Manufacturer	Siemens
Field strength	3T
Model name	Prisma
No. of sessions	1
No. of runs	2 (1 = AP/2 = PA)
Volumes acquired per run	488
Pulse sequence type	Gradient echo, EPI
Field of view	208 × 208 mm
Matrix size	104 × 104 × 72
Slice thickness	2 mm
No. of slices	72
Acquisition orientation	Axial
Whole brain	Yes
Slice acquisition order	Interleaved
TE	37 ms
TR	800 ms
Multi‐band factor	8
Flip angle	75°

#### 2.4.3 Data Preprocessing

Raw functional and anatomical MRI data in NIfTI format were pre‐processed using the default pipeline in CONN toolbox v.22.a (Whitfield‐Gabrieli and Nieto‐Castanon 2012) implemented in MATLAB 2019b (MathWorks Inc., Natick, MA).

#### 2.4.4 Structural Preprocessing

Anatomical T1‐weighted images underwent preprocessing including segmentation into grey matter, white matter, and cerebrospinal fluid (CSF) tissue probability maps using unified segmentation as implemented in SPM12. The images were then normalized to Montreal Neurological Institute (MNI) standard space at 2 mm^3^ isotropic resolution. Grey matter, white matter, and CSF masks were generated for subsequent use in the denoizing procedure.

#### 2.4.5 Functional Preprocessing

Functional data preprocessing began with removal of the initial five volumes to allow for T1 equilibration effects. Slice‐timing correction was applied to account for differences in acquisition time across slices using the middle slice as reference. Head motion was corrected through realignment and unwarp procedures, generating six motion parameters (three translations, three rotations). Outlier identification was performed using Artifact Detection Tools (ART), with outliers defined as volumes with framewise displacement exceeding 0.5 mm or global BOLD signal changes greater than 3 standard deviations. Direct segmentation and normalization to MNI space at 2 mm^3^ isotropic voxels was conducted using parameters derived from the corresponding structural image. Spatial smoothing was applied using an 8 mm full width at half‐maximum (FWHM) Gaussian kernel.

#### 2.4.6 Denoising Procedure

Following spatial preprocessing, additional denoising steps were applied to minimize the influence of non‐neural signals. Linear regression of confounding effects was performed, including BOLD signal from white matter and CSF compartments (five principal components each, using the anatomical CompCor method), six motion parameters and their first‐order temporal derivatives, scrubbing parameters for previously identified outlier volumes, and linear trends. Bandpass filtering between 0.008 and 0.09 Hz was applied to retain frequencies of interest in the BOLD signal. Quality control measures were assessed throughout the preprocessing pipeline, including checks for excessive motion (subjects with more than 20% of their volumes identified as outliers were excluded), registration accuracy, and normalization quality.

### 2.5 fMRI Analyses

#### 2.5.1 ROI‐to‐ROI Functional Connectivity Analysis

Nine ROIs were defined from task‐based activation clusters identified in our previous false feedback study (Patchitt et al. 2025), representing regions that showed significant differential activation during higher versus lower false feedback conditions in this exact sample. The ROIs included the right middle/anterior insula (MNI peak: 41.5, 3.5, 9.5; 130 voxels), the right supramarginal gyrus/opercular cortex/posterior insula (SMGOP1PIR) located at the intersection of somatosensory and interoceptive processing regions (MNI peak: 63.5, −28.5, 21.5; 210 voxels), the right orbitofrontal cortex involved in emotional appraisal and value computation (MNI peak: 49.5, 33.5, −14.5; 125 voxels), a bilateral cerebellum cluster (MNI peaks: 17.5, −60.5, −56.5; 11.5, −62.5, −44.5), a cluster in left cerebellar hemisphere (MNI peak: −18.5, −60.5, −56.5; 127 voxels), the left fusiform/face area (FFA) visual processing region for faces (MNI peak: −36.5, −66.5, −12.5; 250 voxels), the right fusiform gyrus (MNI peak: 41.5, −78.5, −18.5; 84 voxels), the right superior temporal gyrus involved in social cognition (MNI peak: 57.5, −16.5, 5.5; 70 voxels), and the left occipital cortex visual processing region (MNI peak: −12.5, −96.5, −4.5; 156 voxels).

Each region has an established role in the interoceptive and social‐affective processes underlying false feedback susceptibility: insular regions support hierarchical visceral signal processing from primary representation to conscious awareness (Craig 2009; Evrard 2019); the right orbitofrontal cortex integrates interoceptive information with emotional valuation (Rolls 2019); the supramarginal gyrus and opercular cortex support cross‐modal body representation (Keysers et al. 2010); and bilateral cerebellar regions contribute to prediction error computation relevant to mismatches between expected and actual physiological states (Ito 2008; Adamaszek et al. 2017). Together these regions form a theoretically and empirically coherent interoceptive‐emotional system within which resting‐state network topology can be meaningfully interrogated in relation to false feedback susceptibility. This ROI approach offers enhanced statistical power relative to whole brain parcellation schemes and maintains a direct theoretical and empirical connection to the specific interoceptive and emotional processes implicated in false feedback susceptibility.

We nevertheless acknowledge the following limitations of this strategy. First, whilst rest and task architectures have been shown to broadly correspond (Smith et al. 2009; Cole et al. 2014), regions showing task‐evoked activation do not necessarily exhibit coordinated resting‐state functional organization as a network and may not directly recapitulate task‐based co‐activation patterns (Mennes et al. 2013). Second, by restricting graph‐theoretic analyses to connections between the nine selected ROIs, any network effects involving these regions and areas outside this set are necessarily excluded from analysis, potentially omitting topologically important nodes that contribute to false feedback processing. To address this, we also ran a whole‐brain analysis using the full 200‐parcel Schaefer parcellation to examine whether any more distributed effects could be detected. By examining network topology within this empirically defined system, we can test specific hypotheses about how resting‐state functional organization constrains individual differences in processing false physiological feedback. These ROIs were used as nodes for both ROI‐to‐ROI connectivity and graph theory analyses.

For each participant, ROI‐to‐ROI functional connectivity was calculated as the Fisher *z*‐transformed bivariate correlation coefficients between the mean BOLD time series extracted from each pair of ROIs. To investigate the effect of iFFB on functional connectivity patterns, we employed a general linear model controlling for several covariates. The model included iFFB as the main predictor variable of interest while controlling for mean‐centered age, mean‐centered gender, and mean‐centered framewise displacement. The overall group average connectivity was also included in the model. Statistical significance for ROI‐to‐ROI connections was assessed using false discovery rate (FDR) correction at the connection level (*p*_FDR < 0.05).

#### 2.5.2 Graph Theory Analysis

To characterize the topological properties of the functional network defined by our nine ROIs, we conducted a graph theory analysis using the same regions as network nodes using a threshold‐sensitive approach. Further whole brain analyses were conducted using the Shaefer 200 parcellation.

##### 2.5.2.1 Threshold Sensitivity Analysis

Connectivity matrices were thresholded using a proportional approach to maintain equivalent network density across participants. Fixed‐value thresholding does not control for between‐subject variability in overall connectivity strength, resulting in graphs of unequal density. This is a recognized confound for graph‐theoretic metrics and proportional thresholding has therefore been advocated to ensure comparability across individuals (van den Heuvel et al. 2017; Rubinov and Sporns 2010). Given the absence of consensus on any single optimal threshold value (Garrison et al. 2015; Bullmore and Sporns 2009), all analyses were repeated across a range of proportional thresholds (10%–25% in 5% increments), with 15% serving as the primary reported threshold consistent with common practice in the field (van den Heuvel et al. 2017). This range is consistent with the published practice of studies testing proportional threshold ranges of 10%–25% (Hallquist and Hillary 2018). The lower bound avoids node disconnection and unstable path‐length estimates (Rubinov and Sporns 2010), while the upper bound limits inclusion of spurious edges (Váša et al. 2018). Full results across all thresholds are reported in the results Section 3.3.

##### 2.5.2.2 Measure Computation

Thresholded matrices were binarised prior to graph metric computation, such that retained connections were coded as present (1) and all others as absent (0). Binary rather than weighted adjacency matrices were used for three principal reasons. First, the connectivity weights entering the graph pipeline are Fisher *z*‐transformed bivariate Pearson correlation coefficients, which are sensitive to individual differences in BOLD signal‐to‐noise ratio, vascular reactivity, and residual head motion. These are sources of variance that can modulate raw correlation magnitude independently of genuine functional coupling. Retaining graded weights would therefore risk confounding graph metrics with these nuisance factors. Second, the canonical graph metrics calculated are well‐characterized under binary assumptions and have established null‐model benchmarks (Rubinov and Sporns 2010), ensuring comparability with the broader resting‐state graph theory literature (Bullmore and Sporns 2009; van Wijk et al. 2010). Third, our ROI nodes were derived from binarized functional clusters (Patchitt et al. 2025) and span anatomically and physiologically heterogeneous regions; weighted metrics would give disproportionate influence to high‐SNR node pairs, further motivating the binary approach.

##### 2.5.2.3 Group Level Statistics

For each participant and threshold, we calculated several graph metrics including global efficiency, local efficiency, clustering coefficient, characteristic path length, and nodal degree centrality. Network‐based statistics were then computed using the same general linear model as in the ROI‐to‐ROI analysis, with iFFB as the predictor of interest while controlling for the same confounds as before. Statistical significance for graph metrics was determined using non‐parametric permutation testing (5000 permutations) with FDR correction (*p*_FDR < 0.05) to account for multiple comparisons across graph metrics and thresholds. All results were visualized using CONN's built‐in visualization tools and BrainNet Viewer (Xia et al. 2013) for network representation.

##### 2.5.2.4 Analysis Measure Selection

Graph‐theoretic metrics were selected to provide complementary characterization of network properties with direct theoretical relevance to individual differences in false feedback susceptibility. Local efficiency and clustering coefficient index the degree of local specialization within network neighborhoods to reflect the capacity of a region's immediate connections to form densely integrated processing clusters (Latora and Marchiori 2001; Watts and Strogatz 1998). In the context of false feedback, reduced local efficiency within interoceptive regions may reflect degraded local signal integration, potentially compromising the precision of ascending visceral representations and increasing susceptibility to external override by false cardiac information (Barrett and Simmons 2015; Seth and Friston 2016). Global efficiency and characteristic path length index the capacity for rapid information transfer across the wider network (Watts and Strogatz 1998; Achard and Bullmore 2007); given that false feedback susceptibility likely depends on how efficiently interoceptive signals are communicated to higher‐order regions involved in emotional valuation and prediction error resolution, these metrics capture whether the network architecture facilitates or constrains that integration. Nodal degree, betweenness, closeness, and eigenvector centrality collectively characterize each node's topological influence within the network indexing its role as a hub mediating information flow between regions (van den Heuvel and Sporns 2013; Power et al. 2013). Nodes with high centrality may exert disproportionate influence over network‐wide inference, making centrality metrics sensitive to individual differences in how strongly particular regions, such as the posterior insula or orbitofrontal cortex, dominate interoceptive‐emotional integration. Eccentricity, indexing a node's maximum topological distance from all others, was included to characterize the degree to which individual regions are isolated from or embedded within the broader network, with peripheral nodes potentially reflecting reduced access to distributed predictive signals relevant to false feedback resolution (Fornito et al. 2016; Farahani et al. 2019). Together, this metric set was designed to capture distinct but theoretically and empirically complementary aspects of network organization, namely local specialization, global communication, and nodal influence. Each of these measures carries distinct implications for how the resting‐state architecture constrains susceptibility to false physiological feedback.

##### 2.5.2.5 Whole Brain Analysis

To evaluate whether graph‐theoretic findings from the task‐defined ROI analysis were detectable at the scale of the full cortical graph, a whole‐brain sensitivity analysis was conducted using all 200 parcels of the Schaefer cortical atlas with Kong et al. 2021 17‐network assignments (Schaefer et al. 2018; Kong et al. 2021). Graph‐theoretic metrics were computed across the full 200‐node network and tested using the same general linear model as the primary analysis, with iFFB as the predictor of interest and age, gender, and framewise displacement as covariates, across proportional thresholds of 10%, 15%, 20%, and 25%. As an exploratory secondary step, uncorrected statistics were examined from the whole brain analysis with specific attention paid to the Schaefer parcels with greater than five voxels spatial overlap with each of the nine primary task‐defined clusters, to assess directional consistency with the primary ROI findings. These results are reported for transparency and should not be interpreted as independent replications. It should additionally be noted that the Schaefer 200‐parcel atlas is a cortical parcellation derived from surface data and does not include cerebellar parcels (Schaefer et al. 2018). The bilateral cerebellar clusters identified in the primary analysis therefore have no corresponding Schaefer parcels and could not be examined in the whole‐brain sensitivity analysis. The cerebellar local efficiency, clustering coefficient, and betweenness centrality results reported in Section 3.3 consequently remain without atlas‐independent corroboration.

### 2.6 Self‐Report Measures of Interoceptive Awareness

Participants completed the Multidimensional Assessment of Interoceptive Awareness (MAIA; Mehling et al. 2012) questionnaire before the scanning session. The MAIA is a 32‐item self‐report questionnaire assessing eight dimensions of interoceptive body awareness (see Table 3):

**TABLE 3:** Subscales of the multidimensional assessment of interoceptive awareness (MAIA).

Noticing	Awareness of uncomfortable, comfortable, and neutral body sensations
Not distracting	Tendency not to ignore or distract oneself from sensations of pain or discomfort
Not worrying	Tendency not to worry or experience emotional distress with sensations of pain or discomfort
Attention regulation	Ability to sustain and control attention to body sensations
Emotional awareness	Awareness of the connection between body sensations and emotional states
Self‐regulation	Ability to regulate psychological distress by attention to body sensations
Body listening	Active listening to the body for insight
Trusting	Experience of one's body as safe and trustworthy

Items were rated on a 6‐point Likert scale from 0 (never) to 5 (always). MAIA scores were calculated as the mean of all items in the respective subscale.

To explore the relationship between interoceptive awareness and false feedback susceptibility, we conducted Pearson correlations between individual iFFB scores and each MAIA subscale. *p* values were adjusted for multiple comparisons using the false discovery rate (FDR) method. Additionally, we conducted Bayesian correlation analyses to quantify evidence for the null and alternative hypotheses, with Bayes Factors (BF10) interpreted according to standard guidelines (Jeffreys 1961): BF10 < 0.33 indicates substantial evidence for the null hypothesis; 0.33 < BF10 < 3 indicates insensitive or anecdotal evidence; BF10 > 3 indicates substantial evidence for the alternative hypothesis. All analyses were performed using R (version 4.1.0) with the BayesFactor package (Morey and Rouder 2018) and corrplot for visualization.

## 3 Results

### 3.1 Relationship Between iFFB and MAIA Dimensions

iFFB was examined in relation to dimensions of the Multidimensional Assessment of Interoceptive Awareness (MAIA). Correlation analysis revealed modest negative associations across several MAIA dimensions, with Body Listening showing the strongest correlation (*r* = −0.28, *p* = 0.09, BF10 = 1.296). Other subscales including Self‐Regulation (*r* = −0.21, *p* = 0.19, BF10 = 0.748), Attention Regulation (*r* = −0.21, *p* = 0.20, BF10 = 0.729), and Noticing (*r* = −0.20, *p* = 0.21, BF10 = 0.697) demonstrated similar negative trends with iFFB sensitivity, though none reached statistical significance after correction for multiple comparisons. Bayesian analyses indicated mostly anecdotal evidence favoring the null hypothesis, with only Body Listening showing insensitive evidence. While these correlations did not reach statistical significance, the pattern of negative associations suggests that individuals with lower self‐reported body awareness may be more susceptible to false physiological feedback effects.

### 3.2 Graph Theoretical Approach

Graph theoretical analysis of functional network properties revealed several significant associations between network topology and iFFB (See Table 4; Figure 2). We present these findings organized around our core hypotheses regarding interoceptive network organization and false feedback susceptibility. MNI space coordinates can be found in Table 4.

**TABLE 4:** Key findings in relation to our hypotheses, highlighting the patterns of network organization associated with iFFB sensitivity.

Brain region	MNI coordinates (x, y, z) mm	Network property	Effect direction	Beta	Statistics
Finding 1: posterior insula network peripheralization					
SMGOP1PIR	55, −29, 20	Eccentricity	Positive	0.03	*T* (35) = 3.19, *p*‐FDR = 0.027
Finding 2: orbitofrontal cortex functional segregation					
Right orbitofrontal	43, 34, −8	Average path length	Positive	0.01	*T* (33) = 3.25, *p*‐FDR = 0.024
Right orbitofrontal	43, 34, −8	Cost	Negative	−0.01	*T* (35) = −2.72, *p*‐FDR = 0.046
Right orbitofrontal	43, 34, −8	Degree	Negative	−0.06	*T* (35) = −2.72, *p*‐FDR = 0.046
Right orbitofrontal	43, 34, −8	Eigenvector centrality	Negative	−0.01	*T* (35) = −2.76, *p*‐FDR = 0.041
Right orbitofrontal	43, 34, −8	Closeness centrality	Negative	−0.00	*T* (35) = −2.94, *p*‐FDR = 0.027
Finding 3: cerebellar network reconfiguration					
Bilateral cerebellum	0, −70, −47	Local efficiency	Negative	−0.01	*T* (33) = −3.23, *p*‐FDR = 0.005
Bilateral cerebellum	0, −70, −47	Clustering coefficient	Negative	−0.01	*T* (33) = −2.91, *p*‐FDR = 0.029
Bilateral cerebellum	0, −70, −47	Betweenness centrality	Positive	0.00	*T* (35) = 2.80, *p*‐FDR = 0.037
Supporting results: visual processing networks					
Left FFA/FFG	−30, −64, −16	Local efficiency	Negative	−0.01	*T* (34) = −3.17, *p*‐FDR = 0.005
Left FFA/FFG	−30, −64, −16	Clustering coefficient	Negative	−0.01	*T* (34) = −3.30, *p*‐FDR = 0.020
Right fusiform gyrus	30, −77, −15	Local efficiency	Negative	−0.01	*T* (33) = −3.16, *p*‐FDR = 0.005
Right fusiform gyrus	30, −77, −15	Eigenvector centrality	Negative	−0.01	*T* (35) = −2.45, *p*‐FDR = 0.044
Supporting results: network integration					
Left occipital cortex	−15, −93, −9	Local efficiency	Negative	−0.01	*T* (32) = −2.36, *p*‐FDR = 0.028

*Note:* All *p* values are FDR‐corrected for multiple comparisons.

Abbreviations: FFA/FFG = fusiform face area/fusiform gyrus cluster; SMGOP1PIR = supramarginal gyrus, opercular cortex, and posterior insula region cluster.

**FIGURE 2:**
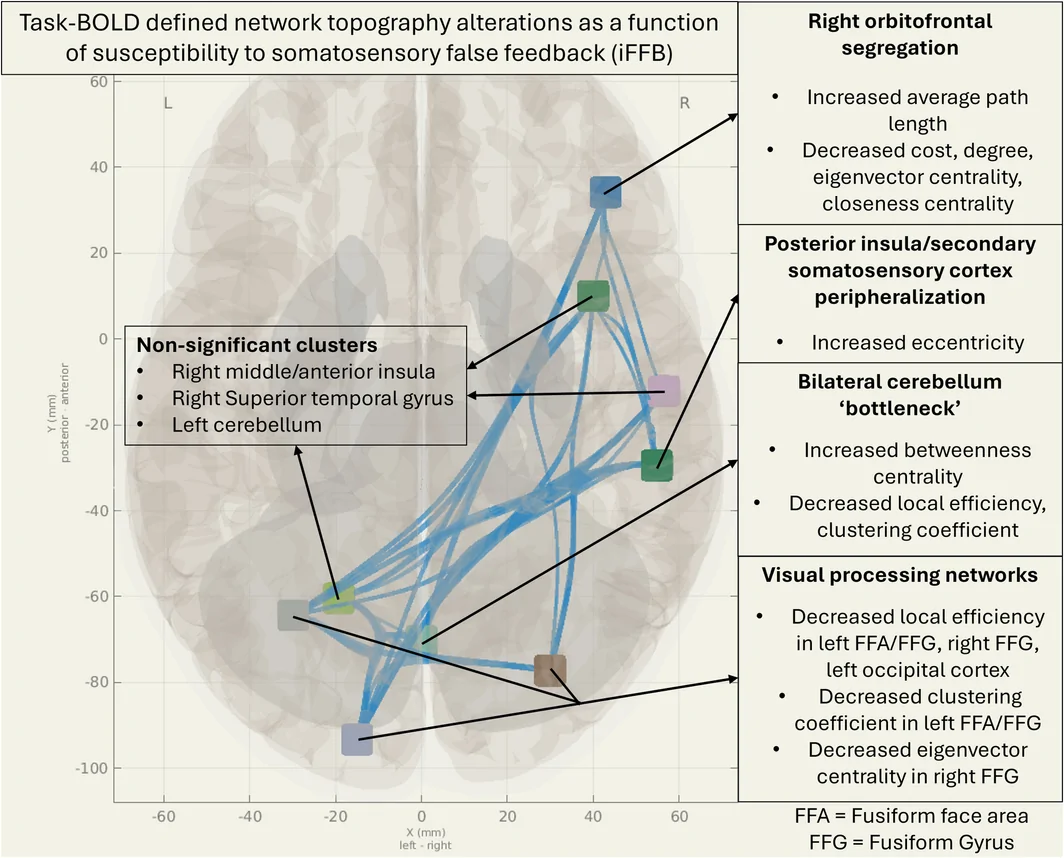
Task‐BOLD defined network topography alterations as a function of susceptibility to somatosensory false feedback (iFFB). Axial view of the nine regions of interest (ROIs) derived from task‐based BOLD activation clusters (Patchitt et al. 2025), displayed on a standard MNI brain template. Nodes represent ROI centroids plotted at their MNI coordinates (x, y axes in mm); blue edges depict functional connections between nodes in the resting‐state network. Four key patterns of network topography were associated with increased iFFB sensitivity. Right orbitofrontal cortex (upper right) showed consistent functional segregation across multiple metrics, including increased average path length and decreased cost, degree, eigenvector centrality, and closeness centrality. The posterior insula/secondary somatosensory cortex cluster (SMGOP1PIR) demonstrated network peripheralization, indexed by increased eccentricity. The bilateral cerebellum cluster exhibited a ‘bottleneck’ pattern characterized by increased betweenness centrality alongside decreased local efficiency and clustering coefficient. Visual processing networks, including left fusiform face area/fusiform gyrus (FFA/FFG), right fusiform gyrus, and left occipital cortex, showed reduced local efficiency; left FFA/FFG additionally demonstrated decreased clustering coefficient, and right FFG showed decreased eigenvector centrality. Three ROIs—right middle/anterior insula, right superior temporal gyrus, and left cerebellum—did not show significant associations with iFFB sensitivity at FDR‐corrected thresholds. FFA = fusiform face area; FFG = fusiform gyrus.

#### 3.2.1 Posterior Insula Network Peripheralization

Individuals more susceptible to false feedback showed significant alterations in the topological position of the posterior insula within the nine‐ROI interoceptive‐emotional network defined from task‐based activations. The supramarginal gyrus/opercular cortex/posterior insula (SMGOP1PIR) cluster showed a significant positive association between eccentricity and iFFB scores (*β* = 0.03, *T* (35) = 3.19, *p*‐FDR = 0.027). Eccentricity, which measures how peripheral a node is within the network (i.e., the maximum shortest path length from that node to any other node in the network), indicates that individuals with a more peripheral posterior insula relative to other false‐feedback‐sensitive regions demonstrate greater sensitivity to false feedback signals.

#### 3.2.2 Orbitofrontal Cortex Functional Segregation

A second key finding was the consistent pattern of right orbitofrontal cortex segregation across multiple network metrics. The right orbitofrontal cortex showed increased average path length (*β* = 0.01, *T* (33) = 3.25, *p*‐FDR = 0.024), alongside decreased network cost (*β* = −0.01, *T* (35) = −2.72, *p*‐FDR = 0.046), reduced node degree (*β* = −0.06, *T* (35) = −2.72, *p*‐FDR = 0.046), reduced eigenvector centrality (*β* = −0.01, *T* (35) = −2.76, *p*‐FDR = 0.041), and decreased closeness centrality (*β* = −0.00, *T* (35) = −2.94, *p*‐FDR = 0.027). These convergent findings indicate that greater segregation of the orbitofrontal cortex from the broader network is associated with increased susceptibility to false feedback effects.

#### 3.2.3 Cerebellar Network Reconfiguration

Our analysis revealed a complex pattern of cerebellar network reconfiguration associated with increased false feedback susceptibility. The bilateral cerebellum cluster showed both reduced local efficiency (*β* = −0.01, *T* (33) = −3.23, *p*‐FDR = 0.005) and reduced clustering coefficient (*β* = −0.01, *T* (33) = −2.91, *p*‐FDR = 0.029), indicating decreased local integration. However, it simultaneously exhibited increased betweenness centrality (*β* = 0.00, *T* (35) = 2.80, *p*‐FDR = 0.037), suggesting it takes on a more critical role as an information hub within the broader network. This finding highlights the cerebellum's role in interoceptive processing and false feedback susceptibility. The pattern suggests that while local processing within the cerebellum is less efficient in individuals more sensitive to false feedback, the cerebellum paradoxically becomes more central to network communication.

#### 3.2.4 Supporting Results

Several additional findings provided convergent evidence for altered network organization in relation to false feedback susceptibility:

##### 3.2.4.1 Visual Processing Networks

The left fusiform face area/fusiform gyrus (FFA/FFG) cluster showed reduced local efficiency (*β* = −0.01, *T* (34) = −3.17, *p*‐FDR = 0.005) and reduced clustering coefficient (*β* = −0.01, *T* (34) = −3.30, *p*‐FDR = 0.020) with increased iFFB scores. Additionally, the right fusiform gyrus demonstrated reduced local efficiency (*β* = −0.01, *T* (33) = −3.16, *p*‐FDR = 0.005) and reduced eigenvector centrality (*β* = −0.01, *T* (35) = −2.45, *p*‐FDR = 0.044). These findings suggest that altered processing within visual regions may contribute to false feedback susceptibility, potentially by affecting how visual emotional stimuli are integrated with interoceptive information.

##### 3.2.4.2 Reduced Network Integration

The left occipital cortex demonstrated a negative association between local efficiency and iFFB sensitivity (*β* = −0.01, *T* (32) = −2.36, *p*‐FDR = 0.028), indicating reduced capacity for local information processing within visual networks.

### 3.3 Threshold Sensitivity Analysis

All FDR‐corrected findings presented above were concentrated at the primary 15% threshold. To further understand the dependence of these results on our chosen threshold, we ran a Graph‐theoretic sensitivity analysis across proportional thresholds (10%, 15%, 20%, and 25%) in the 9 ROI analysis. Results are reported for each node, metric, and threshold (see Table 5).

**TABLE 5:** Threshold sensitivity analysis across proportional thresholds (10%, 15%, 20%, and 25%) in the 9 predefined ROIs of interest.

Region	Measure	0.10	0.15	0.20	0.25
Finding 1: posterior insula network peripheralization					
SMGOP1 PIR	Eccentricity	0.034 (UNC)	0.027	ns	ns
Finding 2: orbitofrontal cortex functional segregation					
Right orbitofrontal	Average path length	0.037 (UNC)	0.024	ns	ns
Right orbitofrontal	Cost	0.015 (UNC)	0.046	ns	ns
Right orbitofrontal	Degree	0.014 (UNC)	0.046	ns	ns
Right orbitofrontal	Eigenvector centrality	0.014 (UNC)	0.041	0.044 (UNC)	ns
Right orbitofrontal	Closeness centrality	0.017 (UNC)	0.027	ns	ns
Finding 3: cerebellar network reconfiguration					
Bilateral cerebellum	Local efficiency	ns	0.005	0.019	ns
Bilateral cerebellum	Clustering coefficient	ns	0.029	0.028	ns
Bilateral cerebellum	Betweenness centrality	ns	0.037	ns	ns
Supporting results: visual processing networks					
LFFA/FFG	Local efficiency	ns	0.005	ns	ns
LFFA/FFG	Clustering coefficient	ns	0.020	0.008 (UNC)	ns
FFGR	Local efficiency	ns	0.005	ns	0.024
FFGR	Eigenvector centrality	0.015 (UNC)	0.044	ns	ns
Supporting results: network integration					
Left occipital	Local efficiency	0.024 (UNC)	0.028	0.023	0.018 (UNC)

*Note:* Values are *p* values, FDR‐corrected for multiple comparisons unless marked (unc) for uncorrected. Cell values are *p* values at the given proportional threshold.

Abbreviations: FFA/FFG, fusiform face area and fusiform gyrus cluster; FFG, fusiform gyrus; ns, not significant; SMG/OP1/PI, supramarginal gyrus, parietal operculum and posterior insula cluster.

Orbitofrontal metrics and SMGOP1PIR eccentricity reach FDR‐corrected significance at 15% only, with uncorrected effects across respective analysis measures/regions at 10% and orbitofrontal eigenvector centrality at 20%, suggesting these effects are expressed most clearly through the strongest and most focal connections in the subnetwork. Cerebellar local efficiency and clustering coefficient survived FDR correction at both 15% and 20%, indicating effects of intermediate connection strength that persist as weaker edges are incorporated, distinguishing these as the most threshold‐stable findings in the analysis. In contrast, fusiform and occipital findings were predominantly restricted to FDR‐corrected significance at 15% except for right fusiform local efficiency, which additionally reached FDR‐corrected significance at 25%, and left occipital local efficiency, which showed uncorrected effects across multiple thresholds but FDR‐corrected significance only at 15% and 20%.

### 3.4 Whole Brain Analysis

No parcel‐level graph metric reached statistical significance after FDR correction at any threshold tested. This null result indicates that the network‐level correlates of false feedback susceptibility are not detectable when the relevant regions are embedded within the broader cortical graph and is consistent with the interpretation that the observed effects reflect organizational properties specific to the interoceptive‐emotional subnetwork rather than a global reorganization of cortical topology. Partial directional support was observed for the SMGOP1PIR eccentricity finding, with two spatially overlapping parcels showing uncorrected effects in the same direction. No directional support was observed for orbitofrontal or visual findings at any threshold (See Table 6).

**TABLE 6:** Graph‐theoretic sensitivity analysis across proportional thresholds (10%, 15%, 20%, and 25%) in the Sheafer 200 whole brain ROI analysis without FDR‐correction.

Shaefer 200 parcel with > 5 overlapping voxels	MNI coordinates	Threshold, *T* value (df), *p*
Right SMGOP1PI cluster (eccentricity)		
17networks_RH_SalVenAttnA_IPL_1	60, −26, 28	ns (at any threshold)
17networks_RH_Aud_ParOper_1	44, −18, 18	0.15, *T* (35) = 1.89, *p*‐unc = 0.033
17networks_RH_SomMotB_4	56, −12, 14	0.20, *T* (35) = 2.23, *p*‐unc = 0.033
Right orbitofrontal cluster (all analysis measures)		
17networks_RH_Language_IFG_1	50, 28, 0	ns (at any threshold)
17networks_RH_ContB_OFC_1	36, 46, −14	ns (at any threshold)
17networks_RH_SalVenAttnB_Ins_1	36, 24, 4	ns (at any threshold)
Right fusiform gyrus cluster (all analysis measures)		
17networks_RH_VisualA_ExStr_3	48, −72, −6	ns (at any threshold)
17networks_RH_VisualA_ExStr_4	29, −68, −12	ns (at any threshold)
Left fusiform gyrus/fusiform face area cluster (all analysis measures)		
17networks_LH_VisualA_ExStr_4	−46, −70, −8	ns (at any threshold)
17networks_LH_VisualA_ExStr_5	−26, −78, −14	ns (at any threshold)
Left occipital lobe (local efficiency)		
17networks_LH_VisualA_ExStr_5	−26, −78, −14	ns (at any threshold)
17networks_LH_VisualC_ExStr_1	−22, −96, 6	ns (at any threshold)
17networks_LH_VisualC_Striate_1	−6, −92, −4	ns (at any threshold)

*Note:* This table represents Schaefer parcels overlapping each of the nine task‐defined clusters by more than five voxels within the wider 200 parcel analysis. Coordinates denote the parcel location in MNI space, in millimeters. No parcel‐level metric survived FDR correction at any threshold.

Abbreviations: ns = not significant; punc = uncorrected *p* value.

## 4 Discussion

Our graph theoretical analysis revealed specific patterns of functional network organization between our pre‐defined interoceptive/FFB regions in covariance with individual susceptibility to somatosensory false physiological feedback (iFFB). These findings provide novel insights into the neurobiological mechanisms underlying interoceptive integration and its influence on emotional processing when presented with false physiological information.

### 4.1 Posterior Insula Eccentricity and Interoceptive Processing

The positive association between SMGOP1PIR (including posterior insula and OP1) eccentricity and iFFB represents one of our most theoretically significant findings. Eccentricity, a graph‐theoretical metric measuring how peripheral a node is within a network (Fornito et al. 2016; Rubinov and Sporns 2010), quantifies the maximum shortest path length from that node to any other node in the network. Higher eccentricity therefore indicates greater peripheralization in relation to the network's core, suggesting reduced efficiency in integrating that region's signals with the rest of the network. The posterior insula and surrounding somatosensory regions serve as a primary cortical target for interoceptive information arriving from thalamic projections (Craig 2009; Critchley and Harrison 2013) and are critical for the representation of true physiological states.

Cytoarchitectonic and functional work, particularly from Evrard and colleagues, has characterized the insula as having specialized substrates organized in a posterior‐to‐anterior structural progression, with posterior granular regions receiving primary interoceptive afferents from the ventromedial thalamus, through dysgranular mid‐insular integration zones, to anterior agranular regions that integrate interoceptive information with broader cognitive and emotional context (Evrard 2019; Craig 2014). This architectural organization supports a hierarchical processing stream wherein posterior regions process primary interoceptive signals and anterior regions integrate these with emotional and cognitive processes (Simmons et al. 2013; Zaki et al. 2012). The increased eccentricity of the posterior insula cluster in relation to false feedback sensitivity indicates that individuals with a more peripheral posterior insula in the functional network demonstrate greater susceptibility to false feedback signals, aligning with Kuehn et al.'s (2016) observation that interoceptive awareness changes posterior insula connectivity profiles.

When the posterior insula and quasi‐interoceptive somatosensory regions occupy a more peripheral position in the functional network, veridical cardiac information may not be sufficiently integrated with other emotional and cognitive processes through the normal posterior‐to‐mid‐to‐anterior insular progression that supports contextual integration of bodily states (Evrard 2019; Craig 2009). Consequently, externally presented false feedback information may more readily override or supplement internal signals, resulting in greater influence on emotional judgments. The peripheralization of this key interoceptive hub suggests that when the posterior insula is less centrally embedded within the broader functional network, the brain may rely more heavily on external sources of physiological information to inform emotional appraisal, making individuals more susceptible to the influence of false feedback.

This contrasts with a finding of decreased connectivity between right posterior and anterior insula while attending to one's own heartbeat versus external cardiac tones (Kuehn et al. 2016). Notably, the finding was significant only in those who performed well in a prior heartbeat counting task, suggesting accuracy‐positive effects during the inhibition of interoceptive information flow along the insula gradient. If heartbeat counting preferentially recruits discriminative somatosensory afferents, momentary down weighting of sub‐conscious/successfully integrated vagal and quasi‐interoceptive afferents would drive, as Kuehn et al. (2016) interpreted, a “selective inhibition of slow signal progression along the posterior‐to‐anterior insula pathway during interoceptive awareness” which would allow “the salient and noiseless detection of one's own heartbeat” through discriminatory pathways. The peripheralized quasi‐interoceptive and posterior insula cortices present in the current investigation remain congruent with the successful integration of veridical interoceptive information under this framework.

Whilst focused connectivity between posterior and anterior insula is negatively correlated with assumed interoceptive ability, posterior insula connectivity to a wider ‘salience network’ is positively correlated with the heartbeat counting task (Chong et al. 2017; Ueno et al. 2020). This is consistent with the present results suggesting that interoceptive processes recruit a loosely hierarchical brain‐wide network and that analyses constrained by conventionally hierarchical models produce differing results (Berntson and Khalsa 2021; Ibáñez and Northoff 2024). Moving beyond simple connectivity strength, the topological position of the posterior insula within broader networks influences susceptibility to false interoceptive information. This provides a more nuanced understanding of how network organization contributes to interoceptive processing and susceptibility to external manipulation.

### 4.2 Orbitofrontal Cortex Segregation and Emotional Appraisal

Multiple network metrics converged to demonstrate altered integration of the right orbitofrontal cortex in relation to false feedback sensitivity. The increased average path length, decreased cost, decreased degree, and reduced eigenvector centrality all indicate greater functional segregation of this region from other network components. The orbitofrontal cortex plays a crucial role in emotional appraisal, value computation, and decision‐making (Coricelli et al. 2005; Rolls 2019).

When the rOFC is more functionally distant from interoceptive and sensory regions, as indicated by these network metrics, the integration of multiple sources of information for emotional appraisal may be compromised. This suggests that individuals with more segregated rOFC functioning may rely more heavily on the most salient available information—in this case, the experimentally manipulated false feedback—rather than integrating it with internal signals and contextual information.

Our finding extends the earlier observation (Ueno et al. 2020) that functional connectivity between the right insula and rOFC correlates with a performance measure of interoceptive sensitivity, extending this relationship to false feedback susceptibility. The consistency of this pattern across multiple network metrics provides strong evidence for the importance of orbitofrontal integration in determining individual differences in false feedback sensitivity.

### 4.3 Network Efficiency and Cerebellar‐Visual Integration

The negative association between local efficiency in cerebellar and visual regions and iFFB indicates that individuals who are more susceptible to false feedback demonstrate less efficient information processing within these regions. From a graph theoretical perspective, reduced local efficiency indicates diminished capacity for specialized processing within local subnetworks (Latora and Marchiori 2001; Rubinov and Sporns 2010). The cerebellum is recognized for its role beyond motor coordination, contributing to cognitive and affective processing (Schmahmann 2019).

The cerebellar cortex is organized into parasagittal modules defined by specific olivo‐cortico‐nuclear connectivity patterns, with each module receiving input from distinct subdivisions of the inferior olive and projecting to specific cerebellar nuclei (Apps and Hawkes 2009). This modular organization, well established through electrophysiological and anatomical tracing studies in animals (Sugihara and Shinoda 2004; Ekerot et al. 1991), forms the basis for functional compartmentalization within the cerebellum. At a macroscopic level, there is established functional topography wherein anterior lobe (lobules I‐V) and lobule VIII subserve sensorimotor functions, while posterior lobe regions (lobules VI‐VII including Crus I and II) participate in cognitive and affective processing (Stoodley and Schmahmann 2009).

Network analyses show that the cerebellum contains distinct functional subnetworks that participate in multiple large‐scale brain networks (notably the salience network) (Buckner et al. 2011; Habas et al. 2009), allowing it to integrate diverse information streams. Our previous work implicated the cerebellum in false feedback processing (Patchitt et al. 2025), and the current findings extend this by demonstrating that the network properties of the cerebellum are critical for determining false feedback susceptibility, consistent with its proposed role in comparing predicted and actual sensory consequences (Ito 2008; Leggio and Molinari 2015).

Notably, the cerebellar cluster showing altered network properties spans bilateral regions including lobules VIIIA and VIIIB, lobule VIIB, left inferior semi‐lunar lobule, and vermis VIIIa. This distribution implicates regions associated with multiple functional domains: lobule VIII is classically associated with sensorimotor processing and forms part of the second motor representation, while also showing activation during interoceptive and emotional tasks (Stoodley and Schmahmann 2010). Lobule VIIB and inferior semi‐lunar lobule are part of the cognitive cerebellum, participating in executive function, working memory, and emotional processing circuits through connections with prefrontal and association cortices (Habas 2021; Guell et al. 2018). The inclusion of vermal regions (particularly vermis VIIIa) is particularly relevant given that posterior vermal damage is associated with affective dysregulation and the limbic component of the cerebellar cognitive affective syndrome (Schmahmann and Sherman 1998). This mixed regional involvement suggests that false feedback susceptibility relates to disrupted integration across cerebellar modules supporting sensorimotor, cognitive, and affective processing—a finding consistent with the multimodal nature of interoceptive integration.

The reduced clustering coefficient in the bilateral cerebellum cluster, right fusiform gyrus, and left fusiform face area/fusiform gyrus cluster further supports this interpretation, indicating decreased local interconnectivity within these networks among individuals more susceptible to false feedback. These parallel findings across multiple network metrics suggest a consistent pattern of reduced local integration in visual‐cerebellar circuits associated with increased sFFB susceptibility.

Interestingly, despite showing reduced local efficiency and clustering, the bilateral cerebellum cluster demonstrated increased betweenness centrality in relation to iFFB. In graph theoretical terms, betweenness centrality quantifies the number of shortest paths between node pairs that pass through a specific node, effectively measuring its importance in controlling information flow (van den Heuvel and Sporns 2013; Power et al. 2013). This suggests that while local processing within the cerebellum is less efficient in individuals more sensitive to sFFB, the cerebellum paradoxically takes on a more critical role as an information hub within the broader network. This pattern of decreased local integration but increased hub‐like properties may reflect a compensatory mechanism (Fornito et al. 2015; Hillary and Grafman 2017) or, alternatively, may indicate that the cerebellum becomes a bottleneck for information processing in these individuals (Mišić et al. 2016; Honey and Sporns 2008), potentially leading to less effective integration of veridical physiological signals with external sensory information. Such network reorganization has been observed in various physiological and pathological states (Bagarinao et al. 2019; Wang et al. 2010), suggesting that alterations in cerebellar network topology may represent a generalizable mechanism for modified interoceptive processing.

### 4.4 MAIA Dimensions and Network Organization in Relation to False Feedback Susceptibility

The pattern of negative correlations between iFFB sensitivity and MAIA dimensions, particularly Body Listening, aligns conceptually with our graph theoretical findings despite not reaching statistical significance. Body Listening, which assesses active listening to the body for insight, showed the strongest negative association with false feedback susceptibility. This complements our primary finding of increased posterior insula eccentricity in individuals more susceptible to false feedback. Both suggest that diminished integration of bodily signals—whether measured through self‐report (MAIA) or functional network topology—may predispose individuals to greater influence from external physiological cues. The modest negative correlations with Attention Regulation and Self‐Regulation further support a framework wherein conscious regulatory aspects of interoception modulate susceptibility to false feedback. While the self‐report and network metrics didn't show strong statistical relationships, their conceptual alignment suggests convergent evidence for interoceptive integration as a key determinant of sFFB effects. These findings extend previous work by Stoica and Depue (2020), who found associations between MAIA dimensions and functional connectivity of regions implicated in our network analysis, suggesting overlapping neural substrates between subjective interoceptive awareness and network‐level organization of interoceptive processing.

### 4.5 Implications for Interoceptive Models and False Feedback Effects

Collectively, our findings support a network‐based model of sFFB susceptibility that emphasizes the importance of functional integration between interoceptive, sensory, and emotional appraisal regions. When these networks are less efficiently integrated (as indicated by reduced local efficiency, increased path length, decreased clustering, and altered eccentricity), individuals appear more susceptible to the influence of false physiological feedback on emotional judgments. This aligns with emerging “small‐world” network perspectives in neuroscience (Watts and Strogatz 1998; Bassett and Bullmore 2017), which emphasize that optimal brain function depends on an appropriate balance between integration and segregation (Sporns 2013; Mišić et al. 2014).

From a predictive processing framework, our results can be interpreted as reflecting altered precision‐weighting of prediction errors in interoceptive systems (Allen et al. 2022; Petzschner et al. 2021; Smith et al. 2017; Barrett and Simmons 2015). While predictive processing models have faced legitimate critiques regarding falsifiability and biological plausibility (Mangalam 2025; Colombo and Wright 2017), the framework offers a useful heuristic for understanding how network topology alterations may influence interoceptive inference. Our empirical findings provide support for core predictions of the model: specifically, that network organization determines how the brain integrates bottom‐up sensory signals with top‐down predictions.

The network topology alterations we observed may impact the relative weighting of internal versus external sources of interoceptive information, consistent with computational accounts of interoceptive inference (Owens et al. 2018; Petzschner et al. 2017; Seth and Friston 2016). Specifically, network metrics indicating reduced integration of veridical interoceptive signals (posterior insula eccentricity) coupled with altered appraisal mechanisms (orbitofrontal segregation) could lead to overweighting of false feedback information in emotional processing.

These results help explain the robust bi‐directional effects observed in sFFB (Patchitt et al. 2025) that were not observed in earlier auditory paradigms (Valins 1966; Gray et al. 2007). The somatosensory modality may more directly engage interoceptive networks, making network topology particularly important for determining the extent to which false feedback influences emotional judgments. Our findings suggest that false feedback effects are not simply the result of a mismatch between veridical and false signals but rather reflect complex network dynamics that determine how interoceptive information is integrated and weighted within emotional processing circuits.

### 4.6 Limitations and Future Directions

Several limitations should be considered when interpreting our findings. First, while FDR correction was applied to our analyses, the exploratory nature of examining multiple graph metrics increases the risk of Type I errors. This concern is particularly relevant in graph theoretical analyses where metrics can be highly interdependent (Zalesky et al. 2010; Fallani et al. 2014).

A further methodological consideration concerns the choice of thresholding approach. Proportional thresholding was adopted to ensure equal network density across participants, addressing the known confound of variable connectivity strength on graph‐theoretic metrics (van den Heuvel et al. 2017; Rubinov and Sporns 2010). This carries particular interpretive complexity in a subnetwork context: unlike whole‐brain graphs, proportional thresholds applied to a nine‐node network mean retained edges represent a much higher proportion of all possible connections, and resulting graphs may not reflect the same topological properties as equivalent densities in whole‐brain analyses (Fornito et al. 2013; van Wijk et al. 2010). Lower thresholds (10%–15%) retain only the strongest, most focal connections, whilst higher thresholds (20%–25%) admit progressively weaker and more distributed edges. Effects specific to lower thresholds therefore reflect strong and focal connectivity relationships, whilst those persisting at higher thresholds are detectable even within a more broadly connected network context (Garrison et al. 2015; Váša et al. 2018). The concentration of FDR‐corrected findings at lower thresholds, and their attenuation as weaker connections are incorporated, suggests that the network‐level correlates of false feedback susceptibility are primarily expressed through the strongest functional couplings within the interoceptive‐emotional subnetwork rather than through its more diffuse connectivity architecture. The distinction between FDR‐corrected and uncorrected findings is particularly important here: effects surviving FDR correction across multiple thresholds represent the most robust evidence in the analysis, whilst findings significant only at uncorrected thresholds, or FDR‐corrected at a single density only, should be interpreted with caution. No findings were significant across the full 10%–25% threshold range at corrected levels, which is consistent with the known sensitivity of graph‐theoretic measures to network density (Garrison et al. 2015; Váša et al. 2018) and reinforces the need for cautious interpretation of threshold‐dependent results.

Finally, the limitations of informing ROI network selection using binarized fMRI BOLD activation clusters warrants consideration when interpreting the data. Regions demonstrating task‐evoked activation do not necessarily function as an integrated resting‐state network, since intrinsic connectivity reflects broader anatomical and functional architecture rather than task co‐activation per se (Mennes et al. 2013). Additionally, restricting analysis to nine ROIs means that path length and eccentricity reflect communication within this subnetwork rather than topological properties of the broader brain network; furthermore, path length in correlation‐based functional networks is derived from inter‐regional correlations in BOLD signal rather than physical axonal connections, and should not be interpreted as equivalent to path length in structural networks (Zalesky et al. 2012).

A whole‐brain sensitivity analysis using all 200 parcels of the Schaefer cortical atlas found no FDR‐corrected significant effects at any threshold tested, indicating that the topological differences associated with false feedback susceptibility are not detectable when the relevant regions are embedded within the full cortical graph. This dissociation suggests that the effects might be localized to the organization of our interoceptive‐emotional clusters, and that a purely whole‐brain approach would have failed to detect them entirely.

Future studies should aim to replicate these findings with confirmatory approaches and larger sample sizes. Additionally, while our graph theoretical approach provides insights into network organization, it does not directly assess causal relationships between network topology and false feedback susceptibility. Causal manipulations using techniques such as transcranial magnetic stimulation or transcranial direct current stimulation targeting key network nodes could help establish causal links (Bassett and Sporns 2017; Santarnecchi et al. 2015). Multimodal approaches combining graph theory with measures of structural connectivity (Damoiseaux and Greicius 2009; Honey et al. 2009) and effective connectivity (Friston et al. 2013; Park and Friston 2013) would provide a more comprehensive understanding of the neural mechanisms underlying false feedback susceptibility. This future research should investigate whether interventions that modulate network connectivity, such as neurofeedback or non‐invasive brain stimulation, can alter susceptibility to false feedback. Specifically, targeting the cerebellar‐visual integration, posterior insula connectivity, or orbitofrontal integration could provide causal evidence for the role of these network properties in false feedback processing. Longitudinal studies examining the stability of these network patterns over time would also provide valuable insights into the trait versus state nature of false feedback susceptibility.

Exploring these network relationships in clinical populations with altered interoceptive processing, such as anxiety disorders or depression, can yield important insights into the role of interoceptive integration in emotional dysregulation. Altered interoception is implicated across psychiatric conditions (Khalsa et al. 2018; Bonaz et al. 2021), understanding how network topology relates to false feedback susceptibility could potentially inform the development of novel therapeutic approaches targeting specific network properties.

## 5 Conclusion

Our graph theoretical analysis demonstrates that individual differences in susceptibility to somatosensory false physiological feedback are associated with specific patterns of functional network organization. Three key patterns emerged: (1) reduced local efficiency and clustering in cerebellar and visual regions alongside increased cerebellar betweenness centrality; (2) increased posterior insula/OP1 eccentricity; and (3) consistent orbitofrontal cortex segregation across multiple network metrics. These results support an interoceptive integration model of false feedback effects and highlight the importance of network topology in determining how individuals weigh and integrate internal and external physiological information during emotional processing.

## Author Contributions

**J. Patchitt:** conceptualization, data curation, formal analysis, investigation, methodology, project administration, software, validation, visualizations, writing – original draft, writing – review and editing. **T. Karapanagiotidis:** formal analysis, writing – review and editing. **S. N. Garfinkel:** funding acquisition, supervision, writing – review and editing. **M. Miller:** writing – review and editing. **M. Tsakiris:** resources, writing – review and editing. **A. Clark:** funding acquisition, resources, writing – review and editing. **H. D. Critchley:** conceptualization, resources, supervision, writing – review and editing.

## Funding

This work was carried out with the support of European Research Council, Synergy Grant (XSCAPE) ERC‐2020‐SyG951631. MT was supported by the European Research Council Consolidator Grant (ERC‐2016‐CoG‐724537) under Horizon 2020 for the INtheSELF project.

## Conflicts of Interest

The authors declare no conflicts of interest.

## Supporting information


**Table S1:** Graph‐theoretic sensitivity analysis across proportional thresholds (10%, 15%, 20%, and 25%) in the Sheafer 200 whole brain ROI analysis without FDR‐correction with specific attention paid to the Schaefer parcels with greater than five voxels spatial overlap with each of the nine primary task‐defined clusters.
**Table S2:** Graph‐theoretic sensitivity analysis across proportional thresholds (10%, 15%, 20%, and 25%) in the 9 ROI analysis. Results are reported for each node, metric, and threshold. Primary analyses were conducted at 15%; results at all thresholds are provided to allow assessment of threshold dependence. FDR‐corrected *p* values are reported where applicable; uncorrected results are indicated.

## Data Availability

The data that support the findings of this study are openly available in Functional Network Organization Underlying Individual at https://osf.io/g59fk/overview.
