# Supplementary material for: Functional Network Organization Underlying Individual Differences in False Feedback Susceptibility: A Graph Theoretical Analysis
Source: Hum Brain Mapp. 2026 Oct 30;47(16):e70640. doi: 10.1002/hbm.70640 (PMC13722775; doi:10.1002/hbm.70640)
Supplement: Supplementary file 1 [file HBM-47-e70640-s001.docx]

Supplementary material

*To evaluate whether graph-theoretic findings from the task-defined ROI analysis were detectable at the scale of the full cortical graph, a whole-brain sensitivity analysis was conducted using all 200 parcels of the Schaefer cortical atlas with Kong 2022 17-network assignments (Schaefer et al., 2018; Kong et al., 2021). Graph-theoretic metrics were computed across the full 200-node network and tested using the same general linear model as the primary analysis, with iFFB as the predictor of interest and age, gender, and framewise displacement as covariates, across proportional thresholds of 10%, 15%, 20%, and 25%. No parcel-level graph metric reached statistical significance after FDR correction at any threshold tested. This null result indicates that the network-level correlates of false feedback susceptibility are not detectable when the relevant regions are embedded within the broader cortical graph and is consistent with the interpretation that the observed effects reflect organisational properties specific to the interoceptive-emotional subnetwork rather than a global reorganisation of cortical topology.*

*As an exploratory secondary step, uncorrected statistics were examined from the whole brain analysis with specific attention paid to the Schaefer parcels with greater than five voxels spatial overlap with each of the nine primary task-defined clusters, to assess directional consistency with the primary ROI findings. These results are reported for transparency and should not be interpreted as independent replications. Partial directional support was observed for the SMGOP1PIR eccentricity finding, with two spatially overlapping parcels showing uncorrected effects in the same direction. No directional support was observed for orbitofrontal or visual findings at any threshold.*

*It should additionally be noted that the Schaefer 200-parcel atlas is a cortical parcellation derived from surface data and does not include cerebellar parcels (Schaefer et al., 2018). The bilateral cerebellar clusters identified in the primary analysis therefore have no corresponding Schaefer parcels and could not be examined in the whole-brain sensitivity analysis. The cerebellar local efficiency, clustering coefficient, and betweenness centrality results reported in Section 3.3.3 consequently remain without atlas-independent corroboration.*

*Supplementary Table 1. Graph-theoretic sensitivity analysis across proportional thresholds (10%, 15%, 20%, 25%) in the Sheafer 200 whole brain ROI analysis without FDR-correction with specific attention paid to the Schaefer parcels with greater than five voxels spatial overlap with each of the nine primary task-defined clusters.*

| *Shaefer 200 parcel with >5 overlapping voxels* | *MNI Coordinates* | *Threshold, T value (df), p value* |
| --- | --- | --- |
| *Right SMGOP1PI cluster (eccentricity)* | | |
| *17networks_RH_SalVenAttnA_IPL_1* | *60, -26, 28* | *ns. (at any threshold)* |
| *17networks_RH_Aud_ParOper_1* | *44, -18, 18* | *0.15, T (35) = 1.89, p-unc = .033* |
| *17networks_RH_SomMotB_4* | *56, -12, 14* | *0.20, T (35) = 2.23, p-unc = .033* |
| *Right orbitofrontal cluster (all analysis measures)* | | |
| *17networks_RH_Language_IFG_1* | *50, 28, 0* | *ns. (at any threshold)* |
| *17networks_RH_ContB_OFC_1* | *36, 46, -14* | *ns. (at any threshold)* |
| *17networks_RH_SalVenAttnB_Ins_1* | *36, 24, 4* | *ns. (at any threshold)* |
| *Right fusiform gyrus cluster (all analysis measures)* | | |
| *17networks_RH_VisualA_ExStr_3* | *48, -72, -6* | *ns. (at any threshold)* |
| *17networks_RH_VisualA_ExStr_4* | *29, -68, -12* | *ns. (at any threshold)* |
| *Left fusiform gyrus/fusiform face area cluster (all analysis measures)* | | |
| *17networks_LH_VisualA_ExStr_4* | *-46, -70, -8* | *ns. (at any threshold)* |
| *17networks_LH_VisualA_ExStr_5* | *-26, -78, -14* | *ns. (at any threshold)* |
| *Left occipital lobe (local efficiency)* | | |
| *17networks_LH_VisualA_ExStr_5* | *-26, -78, -14* | *ns. (at any threshold)* |
| *17networks_LH_VisualC_ExStr_1* | *-22, -96, 6* | *ns. (at any threshold)* |
| *17networks_LH_VisualC_Striate_1* | *-6, -92, -4* | *ns. (at any threshold)* |

*Supplementary Table 2. Graph-theoretic sensitivity analysis across proportional thresholds (10%, 15%, 20%, 25%) in the 9 ROI analysis. Results are reported for each node, metric, and threshold. Primary analyses were conducted at 15%; results at all thresholds are provided to allow assessment of threshold dependence. FDR-corrected p-values are reported where applicable; uncorrected results are indicated.*

| Region | Measure | 0.10 | 0.15 | 0.20 | 0.25 |
| --- | --- | --- | --- | --- | --- |
| SMGOP1 PIR | Eccentricity | **.034 (UNC)** | **.027** | ns. | ns |
|  |  |  |  |  |  |
| OFR | AvPaLen | **.037 (UNC)** | **.024** | ns. | ns. |
| OFR | Cost | **.015 (UNC)** | **.046** | ns. | ns |
| OFR | Degree | **.014 (UNC)** | **.046** | ns. | ns. |
| OFR | EigCen | **.014 (UNC)** | **.041** | **.044 (UNC)** | ns. |
| OFR | CloCen | **.017 (UNC)** | **.027** | ns. | ns. |
|  |  |  |  |  |  |
| BiCerebellum | LocEff | ns. | **.005** | **.019** |  |
| BiCerebellum | ClusCoe | ns. | **.029** | **.028** | ns. |
| BiCerebellum | BetCen | ns. | **.037** | ns. | ns. |
|  |  |  |  |  |  |
| LFFA/FFG | LocEff | Ns. | **.005** | ns. | ns. |
| LFFA/FFG | ClusCoe | Ns. | **.020** | **.008 (UNC)** | ns. |
| FFGR | LocEff | Ns. | **.005** | ns. | **.024** |
| FFGR | EigCen | **.015 (UNC)** | **.044** | ns. | ns. |
|  |  |  |  |  |  |
| OccipitalL | Loc Eff | **.024 (UNC)** | **.028** | **.023** | **.018 (UNC)** |
